# Factors influencing quality of life in patients with active tuberculosis

**DOI:** 10.1186/1477-7525-2-58

**Published:** 2004-10-20

**Authors:** Carlo A Marra, Fawziah Marra, Victoria C Cox, Anita Palepu, J Mark Fitzgerald

**Affiliations:** 1 Faculty of Pharmaceutical Sciences, University of British Columbia, Vancouver, BC, Canada; 2 Centre for Clinical Epidemiology and Evaluation, Vancouver Coastal HealthResearch Institute, Vancouver, BC, Canada; 3 Division of Pharmacy and Vaccines, British Columbia Centre for Disease Control, Vancouver, BC, Canada; 4 Division of Internal Medicine, Faculty of Medicine, University of BritishColumbia, Vancouver, BC, Canada; 5 Centre for Health Outcome and Evaluation Sciences, St. Paul's Hospital, Vancouver, BC, Canada; 6 Division of Respiratory Medicine, Faculty of Medicine, University of British Columbia, Vancouver, BC, Canada

## Abstract

**Background:**

With effective treatment strategies, the focus of tuberculosis (TB) management has shifted from the prevention of mortality to the avoidance of morbidity. As such, there should be an increased focus on quality of life (QoL) experienced by individuals being treated for TB. The objective of our study was to identify areas of QoL that are affected by active TB using focus groups and individual interviews.

**Methods:**

English, Cantonese, and Punjabi-speaking subjects with active TB who were receiving treatment were eligible for recruitment into the study. Gender-based focus group sessions were conducted for the inner city participants but individual interviews were conducted for those who came to the main TB clinic or were hospitalized. Facilitators used open-ended questions and participants were asked to discuss their experiences of being diagnosed with tuberculosis, what impact it had on their lives, issues around adherence to anti-TB medications and information pertaining to their experience with side effects to these medications. All data were audio-recorded, transcribed verbatim, and analyzed using constant comparative analysis.

**Results:**

39 patients with active TB participated. The mean age was 46.2 years (SD 18.4) and 62% were male. Most were Canadian-born being either Caucasian or Aboriginal. Four themes emerged from the focus groups and interviews. The first describes issues related to the diagnosis of tuberculosis and sub-themes were identified as 'symptoms', 'health care provision', and 'emotional impact'. The second theme discusses TB medication factors and the sub-themes identified were 'adverse effects', 'ease of administration', and 'adherence'. The third theme describes social support and functioning issues for the individuals with TB. The fourth theme describes health behavior issues for the individuals with TB and the identified sub-themes were "behavior modification" and "TB knowledge."

**Conclusion:**

Despite the ability to cure TB, there remains a significant impact on QOL. Since much attention is spent on preventative or curative mechanisms, the impact of this condition on QoL is often not considered. Attention to the issues experienced by patients being treated for TB may optimize adherence and treatment success.

## Introduction

Globally, tuberculosis (TB) is a major public health problem [1]. In 1997, the World Health Organization (WHO) estimated that 32% of the world's population was infected with *Mycobacterium tuberculosis *[2]. Tuberculosis was a major cause of morbidity and mortality in Canada early in the 20^th ^Century. However, with the introduction of anti-tuberculosis medications in the 1940's and 1950's, the incidence of TB disease declined significantly [3]. However, after decades of continuous decline in TB rates, it has reached a plateau of 6 per 100,000 population, corresponding to about 2000 cases per year [3,4]. Although this rate of TB disease within Canada in global terms is relatively low, within special high-risk groups, rates exceed those seen in many developing countries. In particular, high rates are seen among Aboriginal persons – both on and off reserve as well as among the foreign born and marginalized inner city populations, especially injection drug users [5-8].

With the development of effective treatment strategies, the focus of TB management has shifted from the prevention of mortality to the avoidance of morbidity. As such, there is increased interest in the quality of life (QoL) experienced by individuals being treated for TB [9]. There are numerous aspects of active TB that may lead to a reduction in QoL. Treatment of active TB requires prolonged therapy (at least 6 months) with multiple, potentially toxic drugs that can lead to adverse reactions in a significant number of patients [10,11]. Also, among foreign born patients, there is considerable social stigma associated with active TB leaving the individual feeling shunned and isolated from their friends and families [12-14]. Finally, among Aboriginal and marginalized inner city populations, there is a lack of knowledge regarding the disease process and its treatment which may contribute to feelings of helplessness and anxiety [15-17].

Few studies have examined quality of life in patients with active TB [18,19]. While these studies determined specific decrements in the QoL in these patients, none have included the mixture of patients (marginalized and foreign born) treated within Canada. Therefore, the objective of our study was to identify areas of QoL that are affected by active TB infection using focus groups and individual interviews [20,21].

## Methods

### Design and Setting

This was a multi-site study involving three TB Centres in Vancouver, British Columbia. Patients were recruited from the TB Clinic at the BC Centre for Disease Control, Willow Chest Pavilion at Vancouver General Hospital and the Downtown TB Clinic. All these clinics are part of the Vancouver Coastal Health Authority. Ethics approval was obtained from the University of British Columbia Behavioural Research Ethics Board and each subject provided signed, informed consent to participate in the study.

### Subjects

Subjects with active TB who were receiving treatment were eligible for recruitment into the study. Subjects who were less than or equal to 16 years of age and who did not speak English, Cantonese or Punjabi were excluded (interpreters for non-English language participants who spoke these languages were available).

### Procedures

The initial contact was be made by the study nurse at the individual clinics. For those individuals residing in the inner-city region of Vancouver, gender-specific focus groups were planned with 6–8 participants who had active TB. Each participant was reimbursed $25 for their time. Focus group discussions are a variation of interviews designed for the purpose of gathering data about a specific topic from a group of individuals. Each focus group was led by an experienced facilitator.

For participants who came to the TB Clinic at the BC Centre for Disease Control (and those who were hospitalized), individual interview sessions were conducted assessing similar information as obtained in the focus groups. The reason for using individual interviews rather than the focus group approach was two-fold. Firstly, as these individuals often had other commitments (such as work or family care), assessments needed to be done at the time of their appointments and could not be specially scheduled. Secondly, due to the cultural backgrounds, most of these participants did not wish to participate in a group setting in which details of their disease and their feelings were explored. Each interview was conducted by an experienced interviewer in combination, when necessary, with an interpreter fluent in Cantonese or Punjabi.

Specifically, in either the focus group or interview setting, the facilitator began the session with an open-ended, standard question that began all sessions (e.g. "how did you find out you had TB?"). Using open-ended, probing questions, participants were asked to discuss their experiences of being diagnosed with tuberculosis, what impact it had on their lives, issues around adherence to anti-TB medications and information pertaining to their experience with side effects to these medications. Each participant was invited to comment on each question and provide their perspective on the content area. At the end of each session, the facilitator summarized salient points that arose during the discussion and invited further comments and discussion around these points and confirmed agreement.

### Data collection and analysis

For all participants, data obtained were audio-recorded, transcribed verbatim, and analyzed. For participants who spoke Cantonese or Punjabi, field notes were kept by the nurse facilitators who are fluent in those languages.

Constant comparative analysis was used as a method to explore and identify patterns and themes that emerged from the data [21]. Various strategies were used to systematically monitor the validity and reliability of the data. Data were analyzed by two individuals experienced with qualitative data and consensus validation was used to confirm categories and the matching of transcribed quotes with categories derived from the analysis. Categories and transcript matching were then reviewed by the focus group facilitator to further ensure that the categories made sense and represented the data they contain. The categories were then collapsed and analyzed for emergent themes.

## Results

We conducted two focus group sessions, one with seven male participants and another six female participants; the rest of the participants for the study underwent individual interviews, including 4 hospitalized patients. In total, 39 persons with active TB participated in the study. The demographics of the study participants are described in Table 1. The mean age was 46.2 years (SD 18.4) and 62% were male. Most of the participants were Canadian-born, either white or Aboriginal, while 38% were foreign-born from South-East Asia, South Asia, Latin America and Africa. The majority of participants were interviewed in English (69%) and the rest required either a Cantonese (18%) or Punjabi (13%) translator. For the majority of patients, concurrent illnesses included HIV, Hepatitis B or C. Thirty-six percent of patients drank alcohol or used illicit drugs on a daily basis. The majority of patients was unemployed with an annual income of ≤$15,999 and a mean level of education of 9.2 years of school (SD 3.1).

**Table 1:** Patient Characteristics

	**Participants (N = 39)**
Mean age, yrs (SD)	46.2 (18.4)
Males, N (%)	24 (62)
Foreign-born, N (%)	15 (38)
Region of origin, N (%)	
Canadian – Caucasian	10 (26)
Canadian – Aboriginal	14 (36)
India/Pakistan	5 (13)
South East Asia	8 (21)
South America	1 (2)
Africa	1 (2)
Language used during interview, N (%)	
English	27 (69)
Cantonese	7 (18)
Punjabi	5 (13)
Interview/focus group session conducted, N (%)	
Outpatient clinic	35 (89)
Hospitalized	4 (11)
Concurrent illness, N (%)	
HIV-positive	12 (31)
Hepatitis B or C	12 (31)
Diabetes mellitus	5 (13)
Cardiovascular disease	3 (8)
Cancer	3 (8)
Epilepsy	1 (2)
Alcohol or recreational drug use, N (%)	
Alcohol	8 (21)
Drugs	6 (15)
Employment status, N (%)	
Full-time	4 (11)
Part-time	5 (13)
Unemployed	20 (50)
Retired	10 (26)
Income	
≤$15,999	35 (89)
$16,000 – $39,999	1 (2)
$40,000 – $49,999	1 (2)
≥$50,000	1 (2)
Years of education, mean (SD)	9.2 (3.1)
Marital status, N (%)	
Single	14 (38)
Married	11 (28)
Common-law	5 (13)
Divorced	8 (21)

Analysis identified four main themes comprising medication related issues, diagnosis, social support and knowledge of TB. The following text provides a summary of the content of the themes with illustrative quotes in Table 2 and 3.

**Table 2:** Selected Illustrative Quotes for Theme 1: Diagnosis Issues for TB

**Theme 1: Diagnosis Issues**
**Sub-Theme: Symptoms**
***Coughing***
"I felt tired all the time and had a cough that just wouldn't go away". (Female)
"I had a really bad cough for 3 months and then I started coughing up blood. This made me scared so I went to the doctor". (Male)
***Fatigue/weakness***
"I just felt tired all the time. I did not have the energy to do anything". (Male)
"I had fatigue and a continuous cough for 6 months. I thought I had persistent flu but then after a while the symptoms got so bad that I went to see a doctor". (Male)
***Fever/nightsweats***
"I had a fever and chest pain for 1 month; I thought this was pneumonia so I went to see my family doctor". (Male)
"I had night sweats for several months and a fever so after a while I went to see my doctor". (Male)
***Asymptomatic***
"I did not know I had TB, I was really surprised because I felt really good". (Male)
"I had a general examination and that's when I found out I had TB, otherwise I had no symptoms" (Female)
**Sub-Theme: Health Care Provision**
***Delayed Diagnosis***
"I had a friend who was sick with TB in the hospital. I asked my GP to get tested but he did not feel I needed to. Anyway, I was negative but I knew something was wrong so I asked for a chest X-ray. He did not agree at the beginning but finally he did and that's when I found that I had TB". (Female)
***Hospitalization***
"I came out of a coma from meningitis and that's when they told me I had TB. They threw me in a TB ward at VGH which was worse then a prison. I didn't like the restrictiveness so I took off...the isolation was too much". (Male)
"The only thing to do at the hospital was to eat and sleep. There are no programs there and you are confined in one area". (Male)
"Everyone wears gloves and masks to come and see you, you feel like a leper". (Male)
**Sub-Theme: Emotional Impact**
***Calm, Accepting, or Apathetic***
"I was okay about it. I knew people who had this before and so I knew I would be in the hospital for a while but then after taking medicines I would be fine". (Male)
"I felt calm and confident in the medical profession". (Male)
***Scared, or Afraid***
"I was scared of dying. My Grandma had it and she was in the sanitorium before she died of it". (Female)
***Shocked/Surprised, or Devastated***
"I was shocked. It was such a surprise because I was working full-time as a nurse in India before immigrating here and I was healthy". (Female)
"I was devastated because I had another illness. I didn't feel that I deserved it". (Female)
***Worried/Concerned or Depressed***
"I was worried about passing it on to other people". (Male)
"I was depressed because I had a daughter whom I could not see while in hospital". (Female)

**Table 3:** Selected Illustrative Quotes for Theme 2: Medication Issues for TB

**Theme 2: Medication Issues**
**Sub-Theme: Adverse Events**
***Gastrointestinal Disturbances***
"I had lots of vomiting after I started taking the pills and didn't have any appetite". (Male)
"I have to eat before I take my pills, if I don't then I feel sick and my stomach hurts". (Male)
***Itchiness***
"I felt itchy all over and was told to take benedryl but that made me really sleepy". (Male)
"I had lots of itchiness when I first started taking the pills but it is better now and I put lotion on my skin". (Female)
"I was so itchy with one of the pills that I could not sleep all night long for days". (Female)
**Sub-Theme: Ease of Administration**
***Size of Medication***
"I felt physically sick because of the size of the pills; they are too big". (Female)
"The pills are so big, it is hard to swallow them". (Female)
"I feel nauseated when I take the pills because they are so large". (Male)
"I can't swallow those white pills; I need to crush then otherwise I vomit it back up". (Female)
***Number of Medications***
"I thought that many tablets a day ...it is not possible to take on an empty stomach". (Male)
" There were too many pills to take at once, especially at the beginning but now it is much better with just six to take in a day". (Female)
**Sub-Theme: Compliance**
***Clinic-based patients***
"I was taking other pills so it was easy to take the TB medications too". (Female)
"I did not forget to take my pills because I want to get better". (Female)
"I understand the importance of taking the tablets so I do not forget; I take them in the mornings, half-hour before my breakfast". (Male)
"I place it in my container the night before so that I remember to take it the next day". (Male)
***Inner-city Patients***
"I always take my pills since I get them with my methadone everyday". (Female)
"The [street] nurse always finds us and gives me the medications". (Male)
"If I've been drinking too much then sometimes I don't know what the time is". (Male)
"If I'm picking empty cans and bottles on the other side of town, it's hard to get to [street nurse name] to get my pills every day". (Male)
"I missed taking some pills because I was drunk or high on drugs". (Female)

### Theme 1: Diagnosis issues

**Figure 1:**
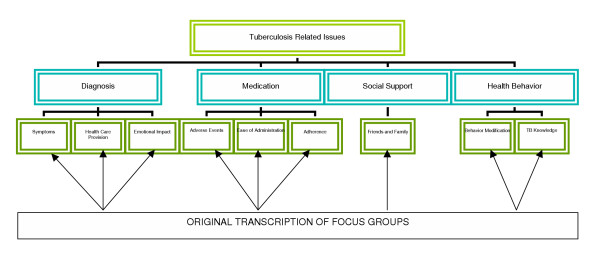
Main themes and sub-themes related to tuberculosis as identified through transcribed focus groups

This theme describes issues related to the diagnosis of tuberculosis (Figure 1). Sub-themes were identified as 'symptoms', 'health care provision', and 'emotional impact'.

#### Symptoms

Thirty-five quotes pertained to symptoms experienced by the participants at the time of diagnosis. Of these, 19 were related to specific symptoms whereas 16 participants expressed the view that they were asymptomatic at the time of being diagnosed. The most common symptoms experienced by the respondents were cough (n = 13), fatigue/weakness (n = 5), fever/night sweats (n = 5) and shortness of breath (n = 4). Most patients sought medical attention due to cough or "pneumonia-like" symptoms and feelings of general malaise. For example, one patient stated "I was coughing up harsh yellow stuff"; while another stated "I started to feel real tired and had a cold that just wouldn't go away".

Four patients expressed that they were "unsure" how they acquired TB. For example, a woman stated "I didn't even know I had it. I was surprised 'cause I felt real good". Other illustrative quotes are shown in Table 2.

#### Health care provision

Most comments, related to the provision of health care at or around the time of diagnosis, were related to community health care providers and their initial hospitalization. Many patients expressed frustration with the health care system at their time of diagnosis due to lack of provider knowledge with respect to tuberculosis. Many patients either felt that they had a delayed diagnosis or delayed treatment due to issues related to their health care provider. For example, on male patient stated, "Family physicians should know more about this disease...where to refer patients to. This is an old disease". Another patient said, "Although my GP gave me a diagnosis, he told me to wait for treatment. We were concerned and phoned the British Columbia Lung Association who referred us to the TB clinic". Another stated, "I contracted a flu-type infection with fever. My GP said go home and take Tylenol but my symptoms continued so I went to see the locum who told me to take Advil. Then I started non-stop coughing. I asked my GP to get an X-ray but he flatly refused."

Many participants reported negative experiences with their initial hospitalization after being diagnosed with TB. Specifically, they stated feelings of isolation, rejection and boredom (Table 2). No participant gave a positive report about the initial hospitalization experience however one 30 year old male participant stated "I had no negative feelings about my hospital stay but it hurt my financial situation...but I knew I had to be there. There are laws against TB."

#### Emotional impact

Thirty-five quotes pertained to emotions experienced by the participants at the time of diagnosis. Of these, patients expressed a wide range of emotions from being calm, accepting or apathetic (n = 11), scared or afraid (n = 7), shocked or surprised (n = 6), "devastated" (n = 4), worried or concerned (n = 4), and depressed (n = 3). Representative quotes for these emotions are presented in Table 2.

Of the individuals expressing apathy or calmness related to the diagnosis, many expressed that TB was just another disease to contend with on top of other chronic conditions. For example, one patient stated "Well, it is like HIV. It is in my system. What can you do?" Another person with terminal cancer stated "I had no reaction to the diagnosis. I am more concerned with the spread of my cancer and that I don't have long to live anyhow".

Of those expressing concern, there were two distinct reasons cited for this emotion: 1) concern for themselves as they knew relatives or friends who had previously been infected with TB and had experience prolonged hospitalization or death; and 2) concern for others in terms of passing the disease on to family and friends. For example, a male patient said "I was kind of scared because the only person I knew who had TB died of it. Also, I was worried about other people catching it from me". Another woman stated "I was scared. It is like an old disease and I know when you have it, it is not very nice to have it, especially because I have a seven month old baby."

The individuals who expressed shock and surprise at the diagnosis attributed these emotions to their lack of symptoms. As such, they had not expected a diagnosis such as TB when they have visited their health care provider despite having other diseases such as HIV (see Table 2).

### Theme 2: Medication issues

This theme discusses the most important factors with respect to medications for the treatment of TB. (Figure 1). As such, the sub-themes identified were "adverse effects", "ease of administration", and "adherence".

#### Adverse events

There were thirty-nine comments related to adverse events experienced by taking the medications. Most of these were related to specific symptoms that were thought to be related to taking specific drug therapies. The most common complaints were related to gastrointestinal disturbances (nausea, vomiting and diarrhea) and itchiness due to isoniazid. Despite having adverse events, patients stated that they continued to take their medications. For example, one female patient said, "There is nothing you can do. You have to just continue". Representative quotes from these participants are included in Table 3.

#### Ease of administration

Most comments related to the dose and dosing schedule pertained to the size (n = 3) and number of tablets/capsules (n = 7). For example, patients felt that the large size of some of the dosage forms (such as ethambutol and rifampin) led to gagging and vomiting. In addition, many patients expressed consternation at the number of pills that they had to take with each dose. For example, one patient said, "When I looked at ten tablets, I thought, on an empty stomach, I cannot". Representative quotes from these participants are included in Table 3.

#### Compliance

Individuals living in the inner city of Vancouver, expressed little concern for compliance-related issues as they either picked up the anti-TB medications with their methadone or the Street-Nurses would find them daily to administer the medications. As an example, one patient stated "It comes with my methadone. When I get that, I get my TB pills". Another stated, "I never worry about it. I know [the Street Nurse's name] will bring it to me". However, despite high compliance in these patients, several identified alcohol (n = 13) or other illicit drug use (n = 8) as being the reason why they had missed doses.

Those who came to the TB treatment clinic expressed high compliance due to the perceived gravity of the diagnosis. For example, one woman patient stated, "It is easy to remember because it is at the fore-front of mind. I want to get rid of it". Another person attributed compliance to the law: "I never forget to take my pills because I don't want to go to jail". Representative quotes from these participants are included in Table 3.

### Theme 3: Social support and functioning

This theme describes social support and functioning issues for the individuals with TB (Figure 1). Specifically, the impact on their relationships with family, friends and peers was affected by TB. In addition, social functioning was impacted through the ability to interact with friends and family as well as engaging in social and leisure pursuits.

Most participants expressed that their family and friends were aware of their TB diagnosis (n = 18), while 11 stated that only their friends knew and 10 stated that only their immediate family knew. Of those who stated that only their friends were aware of their diagnosis, most of these persons residing in the inner city noted that they did not have family with whom they communicated. Representative quotes from these participants are included in Table 4.

**Table 4:** Selected Illustrative Quotes for Theme 3 and 4: Social Support and Health Behavior Issues for TB

**Theme 3: Social Support and Functioning**
**Sub-Theme: Social Support**
***Clinic-based patients***
"My family knows and they comforted me so I felt much better". (Male)
"My wife was calm about it and this gave me support". (Male)
"Mom was concerned for me since her grandmother had died of TB". (Female)
***Inner-city Patients***
"My friends stayed away when they found out, they thought I was contagious. I tried to tell them but still I did not see them again". (Male)
"My friends do no want to hang around me. It's the fear of the unknown...they just know it's airborne and contagious". (Male)
"My partner is okay with it because she has TB too". (Male)
"I don't have any family except my aunt but she was scared to come and see me because she has two children". (Female)
Theme 4: Health Behavior
**Sub-Theme: Behavior Modification**
***Clinic-based patients***
"I run more. I was always a runner. The endorphins help". (Female)
"Vitamins might interact with my medications so I don't take them" (Male)
***Inner-city Patients***
"I eat better... although with my income, this is difficult". (Male)
"This diagnosis was a wake-up call to change my lifestyle. I now eat better and sleep lots" (Male)
"I have been drinking more booze to help manage the side effects of the medications". (Female)
"I drink bottled water and avoid tap water due to my depressed immune system" (Male)
**Sub-Theme: TB Knowledge**
***Clinic-based patients***
"It is important to be cured but you can't get it again" (Female)
"It is very important to get cured...if you aren't cured, you could die" (Male)
"Family doctors should know more about TB. They didn't know what to do with respect to breastfeeding and TB. There really needs to more public education" (Female)
"I don't think I have TB. My doctor told me I have it and now I have to take medications but I am not sure that I really have it" (Male).
***Inner-city Patients***
"Once you get TB, it's in your system" (Male)
"As long as TB can be arrested ...not necessarily cured, that would be OK (Female)"
"People should be more active in spreading the word on the street that TB is still out there...there has to be more outreach programs" (Male)

However, one individual stated that she was "secretive because other people will feel that I am contagious". Another male participant stated a reluctance to tell his friends because "I do not want go cause mass hysteria". A school-age boy did not tell his friends out of fear of being shunned. In addition, he missed eight weeks of school and had to retake several courses. In one instance, fear of being shunned in a Punjabi speaking participant was instilled by the treating physician ("I was told by Dr. [physician's name] that if my community knew, it would empty out the hall [referring to the religious prayer hall]". Another reported that his family "told me not to take my pills anymore because they make me sick" despite being very supportive and understanding regarding the diagnosis. A Cantonese-speaking male stated that "I will be happier once I am cured. Then, I can go out to restaurants and public places again."

There were 39 comments related to the reactions of friends and/or family members to the participants' diagnosis of TB. Of these, most could be categorized as supportive or concerned (n = 21), although others had negative feelings such as fear (n = 7), shock or disbelief (n = 8), and anger (n = 3). One participant stated "my mom was really concerned but my friends did not believe it...they encouraged me to get the right diagnosis". Another stated that her partner had increased his reading on TB and was receiving regular skin tests although her mother and brother would not talk about the TB diagnosis or the clinic visits. Another stated that since his partner was not understanding about modifying his lifestyle, he was forced to end the relationship with her and move out. Representative quotes from these participants are included in Table 4.

### Theme 4: Health behavior

This theme describes health behavior issues for the individuals with TB (Figure 1). Sub-themes were identified as "behavior modification" and "TB knowledge."

#### Behavior modification

When asked if they had done anything else beyond medications to help manage their TB, about half of the participants stated that they had done nothing in particular (n = 16). For example, a Cantonese speaking female stated "I do nothing special as TB is very common". However, of the 12 participants who stated that they had changed their health behavior, seven said that they consumed a healthier diet, four stated that they exercised more, three took vitamin supplements specifically to help their TB, and two used less illicit drugs and alcohol. Representative quotes from these participants are included in Table 4.

#### TB knowledge

In response to the question "do you believe that you will be cured of TB?", most participants (n = 33) stated that they believed that they would be eventually cured. However, some individuals believed that their TB would never be cured ("I believe that I can keep it in remission but it can't be cured") while others were not sure if it could be cured. Two participants denied having TB despite being informed by health care providers and taking medication. There were comments regarding the participants' impressions of provider knowledge of which a representative sample have been included in Table 4.

## Discussion

This qualitative study has revealed that TB has a large impact on affected individuals' QoL through issues related to its diagnosis, treatment, social support and functioning, and health behavior. Specifically, we found that the domains of QoL that were affected by TB included those that are typically affected by most illnesses such as physical functioning and emotional/mental well-being. However, TB patients' social functioning was also affected through isolation, variable social support by family and friends, and the ability to continue with social and leisure activities. Also, the process of getting treatment for TB from the initial hospitalization to the daily medication schedules adversely affected the lives of our participants, although, almost all recognized the need for appropriate treatment.

Although other studies [22,23] have explored patients' attitudes and knowledge regarding TB, we identified only one other study [24] where general health perceptions of patients with TB were investigated. Similar to ours, this study also involved the use of focus groups to elicit areas of QOL affected by TB and many of their results were in general agreement to ours. For example, as with our study, these investigators found that physical functioning, social functioning, and role functioning were all adversely affected by TB. In addition, the participants reported a wide range of psychological reactions including fear, depression and anger. Finally, both studies found a number of comments regarding the difficulties of treatment including those related to the size, number and frequency of dosing of the medications.

However, there were some important differences between our two studies. For example, these investigators included only 10 English speaking patients from the Baltimore city area and 13 health care providers whereas ours included non-English participants through the use of interpreters, a much large sample of patients (n = 39), but no health care providers. In addition, we included hospitalized and ambulatory patients from both inner city and public health clinic environments in order to assess the full spectrum of patients afflicted with this disease. Finally, in our study, all patients had active TB and were receiving treatment at the time of the interviews unlike the Baltimore study who recruited patients who were already cured and had completed treatment. We believe that our methodology of interviewing currently afflicted patients might have minimized recall bias although one potential advantage of the Baltimore approach was determining long-lasting influences of TB on patients lives (the investigators received 17 comments in this regard). Also, the use of health providers in the Baltimore study added an interesting perspective with the provision of comments that were, at times, in direct contrast to those stated by patients with respect to the effects of TB on health related quality of life. For example, most physicians underestimated the impact that TB had on the QoL of patients assuming that, because it was curable, its detrimental effects would be minimal.

These differences in design between the two studies might have accounted for some different findings. For example, the Baltimore study found that the financial well-being of some of the participants was adversely affected through loss of income and health care expenses whereas participants in our study did not report this issue (although this might be attributable to the different health care environments that exist between the two countries in which the studies were conducted or differences in employment status between the two samples). Also, some of the male participants in the Baltimore study reported sexual dysfunction whereas this concern was not reported during our interviews. Finally, patients in the Baltimore study reported spirituality as an important domain which we did not identify as an important theme, perhaps due to the different ethnic/religious make-up of our sample.

One surprising aspect of our results was the negative feelings associated with TB diagnosis and the initial hospitalization. Some participants expressed frustration with their primary care physicians for the lack of a prompt diagnosis or inappropriate management. There was a common perception among many of the participants that health care providers needed more extensive education regarding TB. We have recently commented on the need to consider TB as a diagnosis and in the appropriate setting, consider the initiation of empiric TB treatment [25]. In addition, participants complained of boredom, frustration and isolation with their initial hospitalization. These modifiable factors should be the focus on future improvements in the diagnosis and treatment of TB.

Despite several negative comments regarding the size, dosing schedule and adverse effects of the anti-TB medications, most patients specified that they understood the need for treatment. As such, self-reported compliance was very high and participants reported a variety of different strategies to help manage adverse events. Our inner-city participants expressed gratitude for the street nurses who delivered their medications to them on a regular basis and did not report the intrusiveness and imposition on lifestyle that has been associated with similar programs (such as directly observed therapy or DOT) in other studies [24,26].

Although most comments were related to adverse impacts of TB on QOL, some participants stated that acquiring TB had resulted in positive health behavior modification. Many participants took the development of TB to be a "wake-up" call to change their lifestyle and improve their health behavior by either eliminating or reducing drug and alcohol intake, increasing exercise, or eating better. These findings were also reported by the Baltimore study group suggesting that the positive health behavior impacts of this disease might be widespread throughout those afflicted with TB in North America. Because many of those afflicted with TB in North America engage in other high-risk behaviors such as use of illicit drugs, the overall effects of this health behavior modification might be significant. Future studies should attempt to quantify this impact on the downstream development of other conditions. Although hospitalization for management of TB has negative aspects we have noted that this interlude in subjects with a history of substance abuse allows access to chemical dependency treatment resources while away from their usual chaotic environments.

Although some studies in other countries have shown that TB can result in job loss, participants did not report that this had occurred in our sample [27,28]. One possible reason for this observation could be due to low rate of employment in our sample with only 26% being employed full or part-time.

Our study had some limitations. We examined a self-selected group of TB patients who may not be representative of the entire population in Canada affected by TB. For example, in British Columbia, foreign-born persons account for close to 70% of all TB cases in the province. Despite this, we feel that we were able to get a representative sample of foreign-born persons (almost 40% of our sample was foreign born) as well as a good cross section of marginalized inner city patients [7,29]. In fact, because we attempted to select individuals from different socioeconomic groups (inner city patients vs. those voluntarily attending a public health clinic) and from different ethnic backgrounds (foreign-born, aboriginal-Canadian and other Canadian), we believe that the responses that we received are likely indicative of the areas of QoL which are affected by TB.

## Conclusion

Our study indicates that despite the ability to cure TB with medical therapy, there still remains a sizeable impact on the lives of afflicted patients. Since much of the current attention on TB is spent on preventative or curative mechanisms such as drug therapy, the impact of this condition on QoL is either underestimated or rarely considered. In order to fully evaluate the outcomes that are achieved through TB prevention and treatment, QoL of these patients must be considered. Further studies need to build upon these observations and instruments need to be developed to better characterize QoL in patients with this disease. This process will not only provide an added parameter to evaluate the effectiveness of a given program, but will also focus care providers to be attentive to the non-medication aspects of TB management.

## Authors' contributions

CAM conceived of the study, participated in the design, analysis and co-wrote the initial version of the manuscript. FM conceived of the study, obtained funding, participated in the interviews and focus groups, participated in the analysis, coordinated research staff, and co-wrote the initial version of the manuscript. VC participated in the interviews and focus groups and participated in the analysis. AP participated in the design of the study, and the interviews and focus groups. JMF participated in the design and analysis of the study. All authors read and approved the final manuscript.
